# Supplementary material for: Limited effect of a highway barrier on the genetic structure of a gypsum soil specialist
Source: PeerJ. 2021 Jan 4;9:e10533. doi: 10.7717/peerj.10533 (PMC7789860; doi:10.7717/peerj.10533)
Supplement: Supplemental Information 3 [file peerj-09-10533-s003.docx]

| **Population** | **I.A.M** | **S.M.M** | **T.P.M** |
| --- | --- | --- | --- |
| **1** | 0.00391 | 0.00391 | 0.00391 |
| **2** | 0.00391 | 0.00391 | 0.00391 |
| **3** | 0.00781 | 0.00781 | 0.00781 |
| **4** | 0.00391 | 0.00391 | 0.00391 |
| **5** | 0.00391 | 0.00781 | 0.00391 |
| **6** | 0.00391 | 0.01953 | 0.01953 |
| **7** | 0.00391 | 0.00391 | 0.00391 |
| **8** | 0.00391 | 0.00391 | 0.00391 |
| **9** | 0.00391 | 0.00391 | 0.00391 |
| **10** | 0.00391 | 0.01172 | 0.00781 |
| **11** | 0.00391 | 0.00781 | 0.00781 |
| **12** | 0.00781 | 0.00781 | 0.00781 |
| **13** | 0.00391 | 0.00391 | 0.00391 |
| **14** | 0.00391 | 0.00391 | 0.00391 |
| **15** | 0.00391 | 0.00391 | 0.00391 |
| **16** | 0.00391 | 0.00391 | 0.00391 |
| **17** | 0.00391 | 0.00195 | 0.00391 |
| **18** | 0.00391 | 0.00391 | 0.00391 |
| **19** | 0.00391 | 0.00391 | 0.00391 |
| **20** | 0.00391 | 0.00391 | 0.00391 |
| **21** | 0.00391 | 0.00391 | 0.00391 |
| **23** | 0.00391 | 0.00781 | 0.00391 |
| **24** | 0.00391 | 0.00391 | 0.00391 |
